# Editorial: Multidrug resistant bacteria: new therapeutic approaches for a challenging problem

**DOI:** 10.3389/fphar.2025.1712353

**Published:** 2025-10-14

**Authors:** Gabriela C. Fernández, Younes Smani, Sonia A. Gómez

**Affiliations:** ^1^ Laboratorio de Fisiología de los Procesos Inflamatorios, Instituto de Medicina Experimental (IMEX)-CONICET/Academia Nacional de Medicina de Buenos Aires, CABA, Argentina; ^2^ Andalusian Centro Andaluz de Biología del Desarrollo, Consejo Superior de Investigaciones Científicas (CSIC), Universidad Pablo de Olavide, Seville, Spain; ^3^ Departamento de Biología Molecular e Ingeniería Bioquímica, Universidad Pablo de Olavide, Seville, Spain; ^4^ Servicio Antimicrobianos, Instituto Nacional de Enfermedades Infecciosas (INEI), ANLIS, CABA, Argentina; ^5^ Consejo Nacional de Investigaciones Científicas y Técnicas (CONICET), CABA, Argentina

**Keywords:** antimicrobial resistance (AMR), alternative treatments, nanotechnology, pharmacokinetic modeling, genome-guided combination therapies, antibiotic combinations

The misuse of antimicrobials has accelerated the emergence and spread of multidrug-resistant (MDR) bacterial strains, making infectious diseases increasingly difficult to treat and transforming this issue into a global crisis. Recently, it was estimated that in 2019, 1.3 million deaths were directly attributable to antimicrobial-resistant (AMR) bacterial pathogens (Naghavi et al., 2024). In this context, it is urgent to address critical areas, including the search for effective therapeutic alternatives, rapid diagnostics, and improved surveillance to detect emerging AMR pathogens. In this Research Topic, we have compiled six manuscripts that present novel findings and potential alternatives for the treatment of infections caused by MDR microorganisms.

The article by Pahlevani et al. investigates the development of a potential therapy for ciprofloxacin-resistant *Pseudomonas aeruginosa* (CRP) in burn wound infections. The study evaluates the antimicrobial activity of polyethylene glycol (PEG)-coated ciprofloxacin (CIP)-loaded zeolitic imidazolate framework-8 (ZIF-8) nanozymes (PEG-ZIF-8-CIP) against CRP. *In vitro* assays revealed that PEG-ZIF-8-CIP exhibited superior antimicrobial activity compared to free CIP and ZIF-8 formulations. The nanozymes effectively disrupted CRP biofilms, highlighting their potential to overcome resistance. In a murine burn wounds model, treatment with PEG-ZIF-8-CIP significantly accelerated wound healing, reduced inflammation, enhanced fibroblast proliferation and collagen deposition, and decreased bacterial load in treated wounds, supporting their potential for clinical application. Based on these findings, the authors concluded that PEG-ZIF-8-CIP nanozymes represent a promising dual-action therapeutic approach, combining potent antimicrobial effect with improved wound healing capacity, and may offer an effective treatment for AMR infections in burn patients.

The study by Pasterán et al. addresses the emergence and therapeutic challenges posed by five pan-drug-resistant (PDR) *Klebsiella pneumoniae* isolates recovered from kidney transplant patients in Argentina. The infections were caused by *K. pneumoniae* high-risk clonal group CG258, harboring more than 22 resistance genes, including *bla*_NDM-5_ and a novel variant of *bla*_SHV-231_. The *bla*_SHV-231_ variant carried mutations predicted to reduce susceptibility to aztreonam-avibactam while maintaining its extended-spectrum β-lactamase (ESBL) activity. The authors conducted in-depth genomic analysis of the isolates to characterize the susceptibility profile of *bla*_SHV-231_ and to assess the associated inhibitor resistance phenotype. Successful patients’ treatment was achieved with maximal doses of aztreonam, ceftazidime-avibactam, and oral amoxicillin-clavulanate. The authors conclude that their findings underscore the adaptability of *K. pneumoniae* under therapeutic pressure in clinical settings, emphasizing the importance of rapid phenotypic and genomic characterization of difficult-to-treat resistant bacteria, and suggest that personalized therapeutic strategies can be effective in managing infections caused by PDR pathogens.

The case report presented by Qing et al. describes two pediatric patients with central nervous system (CNS) infections caused by carbapenem-resistant *K. pneumoniae* (CRKP) who were successfully treated with ceftazidime/avibactam (CAZ/AVI). Both children initially received polymyxin B; however, their infections persisted with recurrent fever and abnormal cerebrospinal fluid (CSF) findings. After switching to CAZ/AVI, clinical symptoms rapidly resolved, CSF cultures became negative, and both patients achieved complete recovery without long-term complications or treatment-related adverse effects. This report provides valuable reference data for clinicians managing these challenging infections and underscores the need for larger clinical studies to confirm the efficacy, safety, and pharmacokinetics of CAZ/AVI in pediatric population.

Yang et al. developed a population pharmacokinetic (PopPK) model for polymyxin B (PMB) in critically ill patients with carbapenem-resistant microorganism infections, based on data from 95 patients and 214 blood samples. The model identified optimal dosing regimens of 75 mg and 100 mg administered every 12 h for PMB treatment, with creatinine clearance (CrCL) emerging as key covariate for determining individualized dosing. In addition, the study demonstrated that both CrCL and platelet count (PLT) significantly influenced PMB clearance, highlighting a novel role of PLT in PMB pharmacokinetics that had not been previously reported. Overall, this work provides new insights into PMB pharmacokinetics in critically ill patients and emphasizes the potential for individualized dosing strategies guided by renal function and platelet levels.

The article by Molina Panadero et al. investigates the antibacterial activity of tamoxifen derivatives against methicillin-resistant *Staphylococcus aureus* (MRSA). The study identified 22 tamoxifen derivatives, determined their minimum inhibitory concentrations (MICs), and highlighted three derivatives as the most effective against MRSA. This study underscores the potential of repurposing tamoxifen and its derivatives as novel therapeutic agents to combat antibiotic-resistant *S. aureus*, thereby expanding the range of treatment options in an era of increasing antimicrobial resistance.

The systematic review by Huang et al. examines global trends in AMR in *Enterococcus faecium*, a significant nosocomial pathogen. This review emphasizes the critical public health challenge posed by AMR *E. faecium* and the need for strategic measures to monitor and combat this issue, ensuring that effective treatments remain available for patients. Over recent years, resistance to many antibiotics has gradually increased, although some antibiotics, like gentamicin and chloramphenicol, have shown a decrease in resistance rates. The study confirms that linezolid remains highly effective against *E. faecium*, with a low resistance rate of around 2%. It also brings attention to global disparities in resistance patterns, encouraging tailored approaches to effectively manage and mitigate these challenges.

Collectively, these studies underscore the urgent need for innovative and diversified strategies to combat resistant pathogens. From nanotechnology-based approaches such as PEG-ZIF-8-CIP nanozymes for resistant *P. aeruginosa*, to phenotype and genome-guided combination therapies for PDR *K. pneumoniae*, and pharmacokinetic modeling to optimize PMB dosing, research is advancing toward more precise and effective antimicrobial interventions. The successful use of CAZ/AVI in pediatric CNS infections highlights the potential of novel β-lactam/β-lactamase inhibitor combinations as lifesaving alternatives when traditional regimens fail, while tamoxifen derivatives illustrate the promise of drug repurposing for MRSA. Finally, global surveillance data on *E. faecium* reinforce the importance of continuous monitoring and tailored regional strategies. Together, these findings demonstrate that overcoming the challenge of AMR requires a multifaceted approach integrating new drug development, optimized dosing strategies, innovative therapeutic combinations, and robust global surveillance.
